# The maternal factors associated with infant low birth weight: an umbrella review

**DOI:** 10.1186/s12884-024-06487-y

**Published:** 2024-04-25

**Authors:** Hoda Arabzadeh, Amin Doosti-Irani, Sima Kamkari, Maryam Farhadian, Elahe Elyasi, Younes Mohammadi

**Affiliations:** 1 Department of Epidemiology, School of Public Health, Hamadan University of Medical Sciences, Hamadan, Iran; 2 Research Center for Health Sciences, Hamadan University of Medical Sciences, Hamadan, Iran; 3 Department of Obstetrics and Gynecology, Fatemiyeh Hospital Research Center, Hamadan, Iran; 4 Department of Biostatistics, School of Public Health, Hamadan University of Medical Science, Hamadan, Iran; 5 Modeling of Noncommunicable Diseases Research Center, Hamadan University of Medical Sciences, Hamadan, Iran

**Keywords:** Low birth weight, Periodontal disease, Maternal factors, Umbrella review

## Abstract

**Background:**

In this umbrella review, we systematically evaluated the evidence from meta-analyses and systematic reviews of maternal factors associated with low birth weight.

**Methods:**

PubMed, Scopus, and Web of Science were searched to identify all relevant published studies up to August 2023. We included all meta-analysis studies (based on cohort, case-control, cross-sectional studies) that examined the association between maternal factors (15 risk factors) and risk of LBW, regardless of publication date. A random-effects meta-analysis was conducted to estimate the summary effect size along with the 95% confidence interval (CI), 95% prediction interval, and heterogeneity (I^2^) in all meta-analyses. Hedges’ g was used as the effect size metric. The effects of small studies and excess significance biases were assessed using funnel plots and the Egger’s test, respectively. The methodological quality of the included studies was assessed using the AMSTAR 2 tool.

**Results:**

We included 13 systematic Review with 15 meta-analysis studies in our study based on the inclusion criteria. The following 13 maternal factors were identified as risk factors for low birth weight: crack/cocaine (odds ratio [OR] 2.82, 95% confidence interval [CI] 2.26–3.52), infertility (OR 1.34, 95% CI 1.2–1.48), smoking (OR 2.00, 95% CI 1.76–2.28), periodontal disease (OR 2.41, 95% CI 1.67–3.47), depression (OR 1.84, 95% CI 1.34–2.53), anemia (OR 1.32, 95% CI 1.13–1.55), caffeine/coffee (OR 1.34, 95% CI 1.14–1.57), heavy physical workload (OR 1.87, 95% CI 1.00-3.47), lifting ≥ 11 kg (OR 1.59, 95% CI 1.02–2.48), underweight (OR 1.79, 95% CI 1.20–2.67), alcohol (OR 1.23, 95% CI 1.04–1.46), hypertension (OR 3.90, 95% CI 2.73–5.58), and hypothyroidism (OR 1.40, 95% CI 1.01–1.94). A significant negative association was also reported between antenatal care and low birth weight.

**Conclusions:**

This umbrella review identified drug use (such as crack/cocaine), infertility, smoking, periodontal disease, depression, caffeine and anemia as risk factors for low birth weight in pregnant women. These findings suggest that pregnant women can reduce the risk of low birth weight by maintaining good oral health, eating a healthy diet, managing stress and mental health, and avoiding smoking and drug use.

**Supplementary Information:**

The online version contains supplementary material available at 10.1186/s12884-024-06487-y.

## Introduction

Low birth weight (LBW), defined by the World Health Organization as a weight under 2500 g at birth, stands as a formidable global public health challenge [1–3]. Far beyond a numerical classification, LBW represents a pivotal health metric and a key indicator of intrauterine growth retardation (IUGR). It intricately weaves together the dynamics of fetal development, gestational duration, and birth outcomes, underscoring its significance [4]. LBW contributes to a spectrum of adverse outcomes throughout the life course. Infants with LBW are more susceptible to stunted growth, infectious diseases, neurodevelopmental impairments, compromised cognitive function, and academic performance challenges in childhood and adulthood [5, 6]. In addition to the immediate health consequences, low birth weight can also have long-term effects on a child’s cognitive and physical development. Children born with low birth weight may experience delays in speech, language, and motor skills development, and may be at increased risk of attention deficit hyperactivity disorder (ADHD) and other behavioral problems [7, 8]. LBW is responsible for 60–80% of deaths in the first month of life, LBW significantly heightens the risk of diverse adverse outcomes [1, 9–15]. Moreover, the socioeconomic costs entwined with LBW reverberate across the lifespan, affecting both individuals and society at large [16, 17]. Recognizing the magnitude of health and economic burdens linked with LBW, the World Health Organization has prioritized the reduction of LBW prevalence as a critical public health goal and therefore, sets an ambitious target, aiming for a 30% reduction in prevalence of LBW worldwide between 2012 and 2025 [1, 18]. To attain this ambitious goal, a profound understanding of modifiable determinants of intrauterine growth restriction is essential. This entails a comprehensive exploration of biological, socioeconomic, environmental, and behavioral factors that collectively influence fetal development and contribute to LBW risk [19, 20]. Notably, maternal health conditions and exposures during pregnancy emerge as especially crucial factors amenable to intervention [19, 21–23]. Despite the extensive literature on maternal factors associated with LBW, existing studies yield mixed or inconclusive findings. The absence of a systematic compilation of collective meta-analytic evidence linking various maternal determinants to LBW risk underscores the need for an umbrella review. This unique endeavor is poised to clarify maternal factors with the strongest and most consistent associations with LBW. Its significance lies in elucidating maternal exposures that significantly contribute to LBW risk, thereby informing targeted clinical and public health strategies to address this critical global health issue. Therefore, the primary objective of this umbrella review is to comprehensively synthesize available meta-analytic evidence, with a particular emphasis on evaluating the association between various maternal risk factors and low birth weight (LBW). This meticulous approach ensures a profound exploration of LBW within the intricate context of poor growth and shorter gestation, contributing to a nuanced understanding of this complex public health challenge.

## Method

This umbrella review was conducted and reported by following the PRISMA guideline [24].

### Identifying potential risk factors

In order to pinpoint the potential risk factors associated with Low Birth Weight (LBW), a systematic search was conducted across various online sources to identify all conceivable maternal risk factors linked to LBW. Subsequently, in collaboration with a gynecologist and obstetrician, key risk factors were selected for further investigation in the subsequent phase, which involved locating pertinent meta-analyses.

### Search strategy and eligibility criteria

We conducted a systematic search of PubMed, Scopus, and Web of Science, for meta-analyses published on the association between maternal factors and low birth weight (LBW). The search was conducted on August 2023, without limitations in time, language, and place. We used the following relevant MeSH terms and keywords: Maternal exposure, Smoking, Anemia, Periodontal diseases, Depression, Anxiety, Hypertension, High blood pressure, Body mass index, Quetelet index, Women working, Antenatal care, Alcoholism, Drug use disorders, Caffeine, Thyroid diseases, Infertility female, Infant, Low birth weight, Meta-analysis, Systematic review, Synthesis. The detailed search strategy is included in the supplementary material (Table 1).

**Table 1:** The precise criteria used to stratify the evidence of studies examining maternal risk factors for Low Birth Weight

Evidence	Criteria	factors
Convincing evidence (Class I)	number of cases > 1000 p-value of the meta-analysis < 10^− 6^ I² < 50% 95% prediction interval excluding the null p-value of the egger test > 0.05 and p-value of the Ioannidis test > 0.05	Crack/cocaine Infertility
Highly suggestive (Class II)	number of cases > 1000 p-value of the meta-analysis < 10^− 6^ largest study with a statistically significant effect and class I criteria were not met	antenatal Care smoking
Suggestive (Class III)	number of cases > 1000 p-value of the meta-analysis < 10^− 3^ and class I–II criteria not met	caffeine/coffee anemia Depression Periodontal disease
Weak (Class IV)	p-value of the meta-analysis < 0.05 and class I–III criteria not met	heavy physical workload underweight heavy lifting alcohol hypothyroidism Hypertension
Non-significant Associations (NS)	p-value of the meta-analysis > = 0.05	overweight/obesity

Two authors (HA and EE) independently screened the titles and abstracts of all identified studies and then reviewed the full texts of eligible studies. Disagreements were resolved through discussion with YM. We included all published meta-analyses of cohort, case-control, and cross-sectional studies that examined the association between maternal factors (15 risk factors) and risk of LBW, regardless of publication date. For each factor, we selected the meta-analysis with the highest quality score based on the AMSTAR 2 tool. If two or more meta-analyses had the same quality score, we prioritized the meta-analysis with the latest publication date and the largest sample size.

### Data extraction

Two authors (HA and EE) independently extracted data from the selected studies using a pre-specified form in Microsoft Excel. The extracted data included the following: Factors associated with LBW, First author of the paper, Publication year, Number of participants, Number of studies in the meta-analysis, Study design of included studies, Results of heterogeneity tests, Random effect P-values and the measure of association (e.g., risk ratio, odds ratio) with 95% CI.

### Quality assessment

We assessed the quality of the selected studies using the AMSTAR 2 tool [25]. Any disagreements between the two reviewers were resolved through discussion with a third author (YM). The AMSTAR 2 is a validated and critical appraisal tool for evaluating systematic reviews of randomized trials. It contains 16 items, seven of which are critical items and nine are non-critical items. The rating method was as follows:

Studies with no non-critical items or one non-critical item were defined as high quality.

Studies with more than one non-critical item were defined as medium quality.

Studies with one critical item and with or without non-critical items were defined as low quality.

Studies with more than one critical item with or without non-critical items were defined as critically low quality.

### Statistical analysis

We performed all statistical analyses using the meta-umbrella R package. The meta-umbrella R package is a tool that allows users to perform umbrella reviews with the stratification of evidence. All studies reported odds ratios and relative risks with 95% confidence intervals (CIs) for risk factors of LBW. We used a random-effects model to calculate the pooled odds ratios and relative risks, and we calculated P-values for each risk factor. A significance level of *P* < 0.05 was considered statistical significancy. We assessed heterogeneity among the primary studies using Cochran’s Q test and I2 statistic [26]. The I2 statistic is a measure of the percentage of variation across studies that is due to heterogeneity rather than chance. An I2 statistic of > 50% was considered to indicate high heterogeneity. We also estimated the 95% prediction interval (95% PI) for each risk factor. The 95% PI is a range of values that is likely to contain the value of a single new observation given the specified settings of the predictors [27]. We used Egger’s regression asymmetry test to assess publication bias [28]. Egger’s test is a statistical test that can be used to detect publication bias in meta-analyses. A P-value < 0.05 on Egger’s test was considered to indicate evidence of publication bias. We used Ioannidis test for excess of significance bias to assess the overall bias of the meta-analyses [29]. Ioannidis’ test is a statistical test that can be used to detect bias in meta-analyses, such as selective reporting bias or publication bias. A P-value < 0.05 on Ioannidis test was considered to indicate evidence of overall bias. We also reported the Hedges’ g value for each risk factor. Hedges’ g is a measure of effect size which tells you how much one group differs from another—usually a difference between an experimental group and a control group [30]. Cohen suggested using the following rule of thumb for interpreting results: Small effect (cannot be discerned by the naked eye) = 0.2, Medium Effect = 0.5, Large Effect (can be seen by the naked eye) = 0.8 [31].

### Strength of existing evidence

We used the Ioannidis criteria classification to assess the strength of the evidence of factors for Low Birth Weight. This classification proposes to stratify evidence into five ordinal classes: convincing (class I), highly suggestive (class II), suggestive (class III), weak (class IV), and not significant (ns) (Table 1)

## Results

### Study selection

**Fig. 1:**
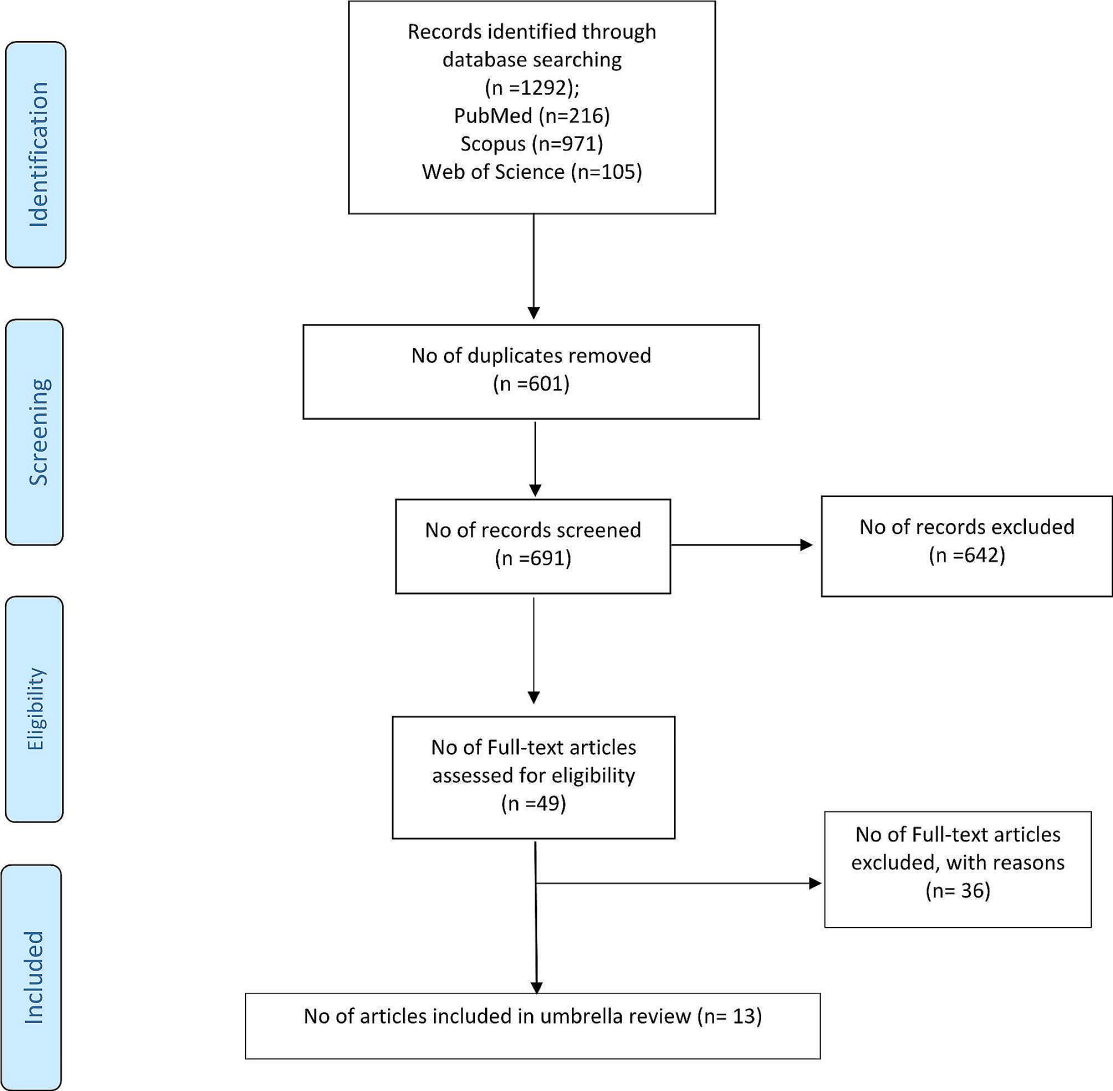
Flowchart of study selection process of the included meta-analyses in umbrella review

The initial database search yielded 1,292 records. After removing 601 duplicates, 691 articles underwent title and abstract screening. A further 49 full-text articles were assessed for eligibility, of which 13 meta-analyses were included in the qualitative synthesis (Fig. 1). The list of excluded studies is provided in Supplementary Table 2.

### Study characteristics

The 13 included meta-analyses comprised a total of 15 separate meta-analyses examining different maternal risk factors for low birth weight (LBW). Collectively, these 15 meta-analyses included 119,358 LBW cases and 5,084,217 participants across 198 individual studies [32–44]. The sample size exceeded 1,000 in all meta-analyses except one (*n* < 500). Publication years they were ranged from 2013 to 2023. The selected meta-analyses included observational study designs including case-control, cohort, and cross-sectional. The meta-analyses examined the following 15 maternal factors about LBW risk: cocaine/crack use, caffeine intake, hypertension, smoking, periodontal disease, depression, anemia, heavy physical workload, heavy lifting, underweight, overweight/obesity, alcohol use, antenatal care, infertility, and hypothyroidism. Detailed characteristics of each meta-analysis are presented in Table 2.

**Table 2:** Characteristics of 15 meta-analyses included in the umbrella review

Risk factors	Reference (year)	No. of total population	No. of studies in meta-analyze	Study design	measure	Summary relative risk estimate (95% CI)	Credibility of evidence	AMSTAR2 quality
crack cocaine	Dos Santo, (2018)	5453	7	Case-control/cohort	OR	2.82 (2.26, 3.52)	Class I	Critically low
Infertility	Carmen ( 2013)	106,040	6	Case-control/cohort	OR	1.34 (1.20, 1.48)	Class I	Critically low
smoking	Pereira, (2017)	3,259,833	30	Case-control/cohort	OR	2.00 (1.76, 2.28)	Class II	Critically low
antenatal Care	Garedew. (2023)	6763	4	Observational follow-up/ Cross-sectional	OR	0.47 (0.37, 0.62)	Class II	Critically low
Periodontal disease	Zhan, (2022)	10,588	14	Case-control/cohort	OR	2.41 (1.67, 3.47)	Class III	Critically low
Depression	Ghimire, (2021)	35,000	12	Case-control/cohort/ cross-sectional	OR	1.84 (1.34, 2.53)	Class III	Critically low
anemia	Rahman, (2016)	237,072	17	Cohort	RR	1.32 (1.13, 1.55)	Class III	Critically low
caffeine/coffee	Feng Jin (2021)	95,612	11	Cohort	RR	1.34 (1.14, 1.57)	Class III	Critically low
heavy physical workload	Cai, (2020)	160,492	5	observational studies	OR	1.87 (1.00, 3.47)	Class IV	Critically low
underweight	Liu, (2019)	313,569	15	Cohort/cross sectional	OR	1.79 (1.20, 2.67)	Class IV	Critically low
lifting ≥ 11 kg	Cai, (2020)	18,158	5	observational studies	OR	1.59 (1.02, 2.48)	Class IV	Critically low
alcohol	Pereira, (2019)	497,023	38	Case-control/cohort	OR	1.23 (1.04, 1.46)	Class IV	Critically low
hypothyroidism	Jiatong (2016)	23,879	5	Cohort	RR	1.40 (1.01, 1.94)	Class IV	Critically low
Hypertension	Getaneh (2020)	1166	14	Case-control/cohort/ cross-sectional	OR	3.90 (2.73, 5.58)	Class IV	Critically low
overweight/obesity	Liu, (2019)	313,569	15	Cohort/cross sectional	OR	1.17 (1.00, 1.36)	NS	Critically low

### Methodological quality

The methodological quality of all included meta-analyses was rated as “critically low” based on the AMSTAR-2 tool. Individual quality assessment scores are provided in Supplementary file, Table 3.

### Summary effect sizes

Of the 15 maternal factors examined, 13 demonstrated statistically significant associations with LBW risk at *p* ≤ 0.05 and were considered as risk factors: periodontal disease, depression, smoking, hypertension, cocaine/crack use, anemia, heavy physical workload, heavy lifting, underweight, alcohol use, hypothyroidism, caffeine intake, and infertility. One factor, antenatal care, showed a significant protective effect against LBW (*p* < 0.001). Overweight/obesity was not significantly associated with LBW (*p* = 0.054).

**Fig. 2:**
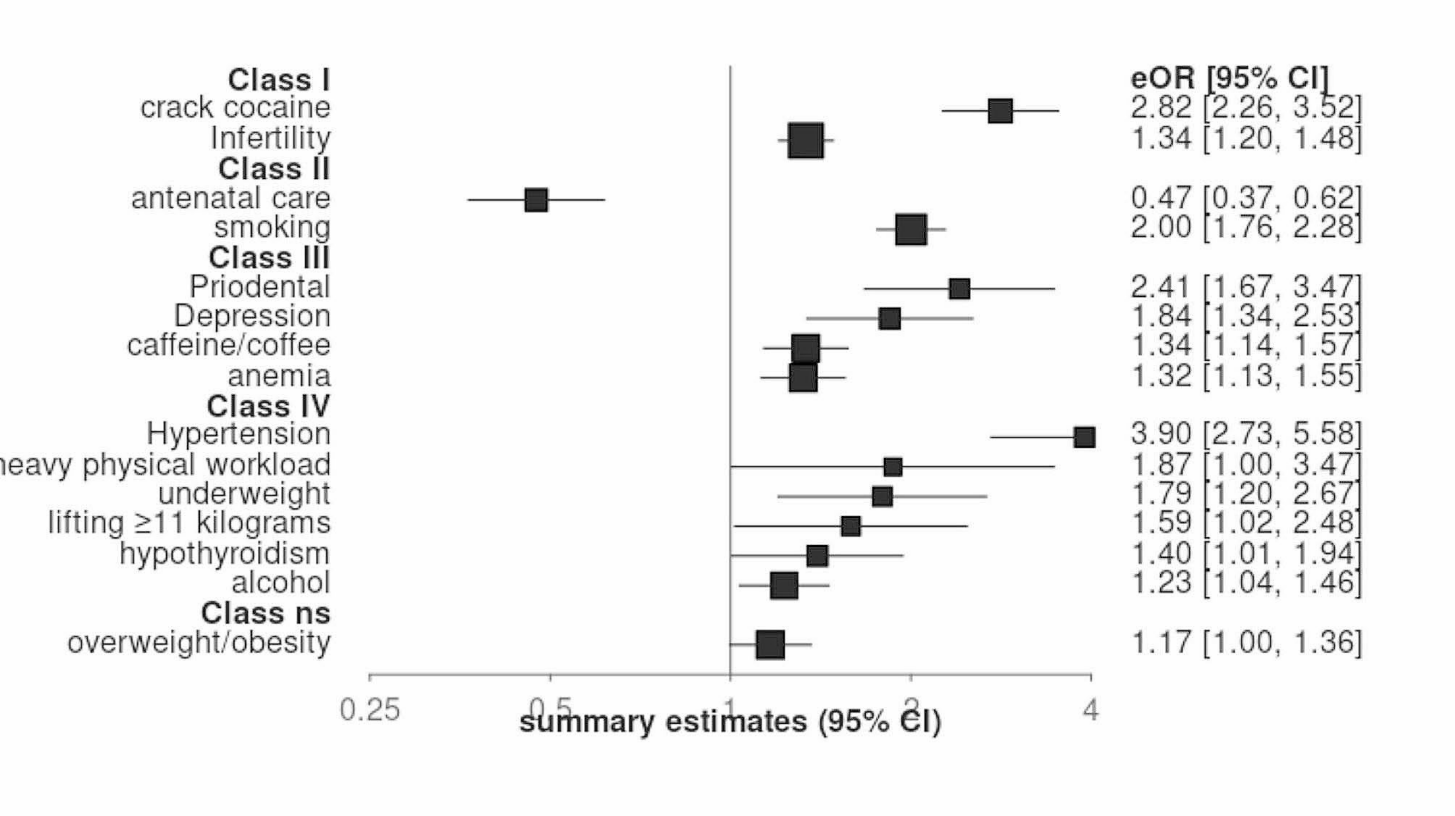
Summary estimates with 95% confidence intervals from 15 associations for LBW

Based on the evaluation of effect size and evidence strength, cocaine/crack use (OR 2.82, 95% CI: 2.26–3.52) and infertility (OR 1.33, 95% CI: 1.2–1.48) were classified as convincing risk factors (Class I). Antenatal care (OR 0.47, 95% CI: 0.36–0.61) and smoking (OR 2.00, 95% CI: 1.75–2.28) showed highly suggestive evidence (Class II). Periodontal disease, depression, anemia, and caffeine intake were considered suggestive evidence (Class III). The remaining risk factors demonstrated weak evidence (Class IV). Summary effect sizes and evidence grades are presented in Table 3; Fig. 2.

**Table 3:** The credibility of the evidence for included meta-analyses

Risk factors	Random effects P-value	Number of cases	Excess of significance bias (p-value) ESB	the value of the pooled effect size expressed in equivalent Hedges’ g (eG)	small-study effects (p-value for Egger	Prediction Intervals	Largest study (95% CI)	Heterogeneity (I2%)	Classification
crack cocaine	< 0.001	1072	0.435	0.572	0.645	1.699–4.689	2.098–3.228	25.433	Class I
Infertility	< 0.001	7770	0.629	0.16	0.816	1.148, 1.556	0.769, 2.1	0	Class I
smoking	< 0.001	15,413	0.443	0.383	0.495	1.131–3.543	1.251–1.75	66.136	Class II
antenatal Care	< 0.001	1211	0.882	0.411	0.481	0.586–1.339	0.329–0.489	55.79	Class II
Periodontal	< 0.001	7202	0.002	0.485	0.006	0.599–9.702	0.891–1.561	81.892	Class III
Depression	< 0.001	6244	< 0.001	0.338	0.01	0.638–5.337	1.224–2.313	81.275	Class III
anemia	< 0.001	9394	< 0.001	0.154	0.02	0.752–2.324	1.15–7.62	65.679	Class III
caffeine/coffee	< 0.001	5072	0.246	0.16	0.071	0.872, 2.046	1.017, 2.018	52.90	Class III
heavy physical workload	0.048	12,794	0.014	0.344	0.05	0.225–15.494	2.122–3.432	91.388	Class IV
underweight	0.002	9625	0.12	0.29	0.147	0.433–6.621	1.085–1.336	86.144	Class IV
lifting ≥ 11 kg	0.042	1891	0.8	0.255	0.253	0.323–7.808	1.302–4.421	81.955	Class IV
alcohol	0.001	30,444	0.004	0.114	0.619	0.48–3.146	0.581–1.579	89.166	Class IV
hypothyroidism	0.045	1122	0.468	0.184	0.603	0.66, 2.955	0.819, 1.76	21.53	Class IV
Hypertension	< 0.001	479	0.253	0.75	0.924	1.083, 14.038	1.789, 40.869	75.07	Class IV
overweight/obesity	0.054	9625	0.005	0.084	0.334	0.733–1.854	1.303–1.722	63.421	NS

### Heterogeneity and bias

Across the 15 associations, 12 (80%) exhibited high heterogeneity (I2 > 50%) and three (20%) had low heterogeneity (I2 < 50%). The 95% prediction interval included the null value for 11 associations (73%) and excluded the null for four associations (27%). Small study bias was indicated in three meta-analyses based on significant Egger’s tests (*p* < 0.05) for periodontal disease, depression, and anemia. No evidence of excess significance bias was found for 10 factors according to Ioannidis testing; the remaining five showed potential excess significance bias. According to Hedges’ g values, effect sizes were small for seven factors, medium for six factors, and large for two factors. Detailed results for heterogeneity and bias assessments are presented in Table 3.

## Discussion

This umbrella review aimed to examine the connection between maternal factors and the risk of low birth weight (LBW). We analyzed 15 maternal factors based on 13 systematic review and meta-analysis studies involving 119,358 LBW cases. Our findings revealed that periodontal disease, anemia, depression, hypertension, hypothyroidism, infertility, underweight, heavy physical workload, lifting ≥ 11 kg, smoking and alcohol, caffeine, and crack/cocaine use during pregnancy were identified as risk factors for LBW. Additionally, antenatal care was found to be a protective factor against LBW.

Our study found highly suggestive evidence that antenatal care can decrease the risk of low birth weight. Specifically, our results showed that mothers who received prenatal care at least once during pregnancy had a lower risk of LBW in their infants [44]. Furthermore, we did not identify significant publication bias or excess significant bias (publication bias) in our study. Our findings are consistent with other studies [45–47], which support the notion that promoting prenatal care for pregnant women can serve as a valuable and essential strategy to enhance newborn health outcomes and mitigate the risk of LBW.

Our study provides convincing evidence that infertility is a risk factor for low birth weight (LBW). In our study, infertility was defined as pregnancy occurring after 12 months of trying [48, 49]. Our results are consistent with a published meta-analysis, which found that twins conceived through in vitro fertilization (IVF) have a higher risk of LBW [50]. Given that various underlying pathologies can lead to infertility, some of these mechanisms may also contribute to adverse pregnancy outcomes [51, 52].

Our study found suggestive evidence that smoking, caffeine intake, and narcotics such as crack/cocaine use during pregnancy are risk factors for low birth weight (LBW). Additionally, we found weak evidence that alcohol can increase the risk of LBW. Previous studies have attributed the negative effects of smoking on LBW to nicotine, which affects the cardiovascular system of the mother, leading to tachycardia and peripheral vasoconstriction [53–55]. This results in hypoxia and low levels of nutrients delivered to the placenta, ultimately causing fetal growth restriction [56, 57]. Alcohol and crack/cocaine use can also interfere with the transfer of important nutrients for growth by the placenta and affect the fetus’s ability to receive sufficient oxygen and nourishment, leading to LBW [56, 58, 59].

However, in the included meta-analysis that examined the effect of alcohol on LBW, high heterogeneity and publication bias were reported, and it was classified as weak evidence. This heterogeneity may be due to differences in methodology, sample size, and specificities. Therefore, we must be careful when interpreting the association between alcohol and LBW. Caffeine metabolism is slower in pregnant women, and caffeine can easily be transmitted across the placenta due to its presence in amniotic fluid, umbilical cord, urine, and plasma. The fetus cannot produce enough enzymes for caffeine metabolism due to liver immaturity, leading to an increased risk of LBW [60]. Previous dose-response meta-analyses have shown that there is a graded relationship between caffeine consumption and LBW, with every 100 mg of maternal caffeine consumption per day (about one cup of coffee) increasing the risk of LBW [61, 62]. However, evidence from previous dose-response meta-analyses has also shown that there is no identifiable threshold for caffeine intake to be a risk factor for adverse pregnancy outcomes such as LBW [63].

Our umbrella review found that diseases such as hypertension, periodontitis, depression, anemia, and hypothyroidism are associated with an increased risk of low birth weight (LBW). The study by Rahman et al. demonstrated that pregnancy-induced hypertension is an independent risk factor for LBW, In the results of a WHO secondary analysis survey conducted in low- and middle-income countries, pregnancy with hypertension was associated with a double risk of LBW [64, 65]. Our meta-analysis showed that almost one-third of pregnancies with hypertension result in the birth of LBW infants, although the results may have been influenced by several confounders and high heterogeneity (I2 = 75%), However, another meta-analysis study conducted on cohort studies yielded results that are consistent with our findings [66].

Depression during pregnancy has been associated with poorer maternal health behaviors, such as unhealthy diet, physical weakness, poverty, unhealthy lifestyle, and smoking, which could increase the risk of LBW [21, 67]. A meta-analysis published in 2017 indicated a decrease in maternal hemoglobin level during the first pregnancy is significantly related to the risk of LBW, although there was no significant relationship with the second and third trimesters [68]. Periodontitis can directly cause infection of the placenta and fetus through periodontal bacteria, and several published studies have confirmed that periodontal disease is significantly associated with adverse pregnancy outcomes [69–71]. However, a case-control study showed that periodontal disease is not significantly associated with LBW, even after controlling for potential confounders [72], which may be due to recall bias or selection bias.

Thyroid disorders such as hypothyroidism in pregnant women can be a risk factor for LBW because thyroid hormone regulates fetal growth and development throughout pregnancy. The fetus needs placental hormone transfer from the mother to access thyroid hormone, especially during the first 18 to 20 weeks of gestation [73]. It is important to note that I2 > 65 was reported in these four meta-analyses, and publication bias and publication bias were significant in periodontal diseases, depression, and anemia. However, these factors were classified as suggestive evidence (class III), while hypertension was placed in the category of weak evidence despite having an OR of 3.8. These points should be considered when interpreting these associations.

Over the past few decades, there has been an increase in the proportion of working women. However, past studies have shown that physical work can be a factor for adverse pregnancy outcomes, including low birth weight and preterm delivery. Heavy physical activity can lead to the contraction of the uterus and increase the risk of premature labor by increasing the level of noradrenaline [43, 74, 75].

The meta-analysis we reviewed showed a positive and significant correlation between heavy physical workload, lifting ≥ 11 kg, and low birth weight. However, this meta-analysis had high heterogeneity (> 80) and was classified as weak evidence (class IV). Significant excess bias was also reported for lifting ≥ 11 kg. Other meta-analysis studies have shown that long working hours compared to standard hours and standing at work for > 4 h per day during pregnancy are associated with an increased risk of miscarriage, low birth weight, and preterm delivery [76, 77].

Several previously published studies have demonstrated that maternal body mass index (BMI) during pre-pregnancy or early pregnancy can affect the health of both mothers and infants [78–81]. In our investigation, we found that maternal underweight was positively and significantly related to low birth weight, while the relationship between obesity/overweight and low birth weight was not significant. Another meta-analysis, which was based on cohort studies and adjusted for confounders, supports our claim that maternal underweight is a risk factor for low birth weight in infants [82].

### Implication

Given the significance of understanding the determinants influencing low birth weight to mitigate and diminish this unfavorable pregnancy outcome, our findings hold the potential to assist policymakers and healthcare practitioners in sustaining and enhancing the well-being of both mothers and infants. Various recommendations for prospective research endeavors can be proposed, including the imperative to undertake meta-analyses on unexplored factors impacting low birth weight, such as infectious diseases, which were not investigated in our study.

### Strengths and limitations

An umbrella review serves as a comprehensive document that provides a useful overview of reviews on a specific topic, including all relevant reviews [83–85], Our study represents the first umbrella review conducted on risk factors for low birth weight (LBW). Additionally, significant publication bias, as determined by Egger’s test, was observed in only three meta-analyses (Periodontal Disease, Depression, Anemia), while most of the others did not report significant publication bias.

However, it is important to acknowledge the limitations of our study when interpreting the findings. Firstly, the search was limited to three databases (PubMed, Scopus, and Web of Sciences), potentially introducing selection bias despite their extensive coverage. Secondly, we focused solely on the association between certain maternal factors and the risk of LBW, neglecting other potential factors that may contribute to LBW risk. Thirdly, some of the included meta-analyses exhibited high heterogeneity, and the underlying factors contributing to this heterogeneity (such as age, gender, nationality, and study region) were not further explored, which may have influenced our results. Lastly, the validity of umbrella reviews relies on the quality of the included meta-analyses, and since all the meta-analyses in our study were of low quality, we express concern regarding the robustness of our findings.

It is important to acknowledge that certain risk factors, such as periodontal disease, may have been subject to varying degrees of scrutiny in the literature, with some more rigorous studies suggesting limited impact on outcomes. While our review accurately reflects the existing evidence, it is essential to note the nuanced nature of certain risk factors and their potential influence on outcomes. Additionally, concerns have been raised about the analyses of cocaine effects, with criticisms centered on the adequacy of controlling associated risk factors. Recognizing these nuances is crucial in interpreting our findings, and future research endeavors may benefit from addressing these concerns and incorporating diverse study designs for a more comprehensive understanding of the complex interplay between risk factors and outcomes.

## Conclusions

This umbrella review aimed to systematically and comprehensively collect available data from published meta-analyses that investigated the association between maternal factors and the risk of low birth weight. The goal was to provide clinical decision-makers and researchers with a robust evaluation of these associations, with the ultimate aim of preserving and improving the health of mothers and babies and preventing low birth weight.

Our findings suggest that periodontal disease, anemia, depression, infertility, smoking, and substance use (such as crack/cocaine) during pregnancy are associated with an increased risk of low birth weight, supported by suggestive evidence.

### Electronic supplementary material

Below is the link to the electronic supplementary material.

Supplementary Material 1

Supplementary Material 2

## Data Availability

No datasets were generated or analysed during the current study.
