# Supplementary material for: The maternal factors associated with infant low birth weight: an umbrella review
Source: BMC Pregnancy Childbirth. 2024 Apr 25;24:316. doi: 10.1186/s12884-024-06487-y (PMC11044292; doi:10.1186/s12884-024-06487-y)
Supplement: Supplementary file 2 — Supplementary Material 2 [file 12884_2024_6487_MOESM2_ESM.docx]

**Table 1. Search strategy for retrieving relevant meta-analysis studies**

1. “Maternal Exposure” OR “Maternal factor”
2. Smoking or “Behavior Smoking” or “Smoking Habit” or "Cigarette Smoking” or "Cigar Smoking”
3. Anemia
4. “Periodontal Diseases” or Parodontosis or Pyorrhea Alveolaris
5. Depression or Anxiety
6. Hypertension or “Blood Pressure, High” or “Pre-Eclampsia”
7. "Body Mass Index"
8. “Prenatal Care” or “Antenatal Care”
9. Alcoholism or ” Alcohol Dependence”
10. “Substance-Related Disorders” or “Drug Use Disorders” or “Substance Abuse” or “Substance Addiction” or “Drug Abuse”
11. “Women, Working”
12. Caffeine or Vivarin
13. “Thyroid Diseases” or Hypothyroidism or Hyperthyroidism
14. "Infertility, Female"
15. #1 or #2 or #3 or #4 or #5 or #6 or #7 or #8 or #9 or #10 or #11 or #12 or #13 or #14
16. Infant, Newborn OR Newborn OR Neonate
17. Infant, Low Birth Weight OR Low Birth Weight
18. Systematic review OR Meta-analysis OR Meta analysis OR synthesis
19. #15 and #16 and #17 and #18

Table 2. The excluded systematic reviews and meta-analyses, along with the reasons.

| Author | Risk factor | Reason |
| --- | --- | --- |
| McDonald et al. (1) | BMI | lower quality or smaller sample size |
| Rahman et al. (2) | BMI | lower quality or smaller sample size |
| Zhen et al. (3) | BMI | lower quality or smaller sample size |
| Liu et al. (4) | Antenatal care | lower quality |
| Zheng et al. (5) | smoking | lower quality |
| Palmer et al. (6) | Working | smaller sample size |
| Casas et al. (7) | Working | smaller sample size |
| Cai et al. (8) | Working | smaller sample size |
| Jarde et al. (9) | Depression | lower quality |
| Huang et al. (10) | Depression | lower quality |
| Grote et al. (11) | Depression | lower quality |
| Grigoriadis et al. (12) | Depression | lower quality |
| Fecadu et al. (13) | Depression | lower quality or smaller sample size |
| Ding et al. (14) | Depression | lower quality |
| Dadi et al. (15) | Depression | lower quality or smaller sample size |
| Liu et al. (16) | Depression | lower quality |
| Grigoriadis et al. (16) | Depression | lower quality |
| Patra et al. (17) | alcohol | smaller sample size |
| Haider et al. (18) | Anemia | smaller sample size |
| Figueiredo et al. (19) | Anemia | lower quality |
| Vergnes et al. (20) | Periodontal disease | Low Birthweight was risk factor |
| Konopka et al. (21) | Periodontal disease | smaller sample size |
| Khader et al. (22) | Periodontal disease | lower quality or smaller sample size |
| Corbella et al. (23) | Periodontal disease | lower quality or smaller sample size |
| Corbella et al. (24) | Periodontal disease | lower quality or smaller sample size |
| Porto et al. (25) | Periodontal disease | lower quality |
| Polyzos et al. (26) | Periodontal disease | smaller sample size |
| Derakhshan et al. (27) | hypothyroidism | lower quality |
| LUTIGER et al. (28) | Drug abuse | lower quality or smaller sample size |
| Hulse et al. (29) | Drug abuse | lower quality or smaller sample size |
| Addis et al. (30) | Drug abuse | lower quality or smaller sample size |
| Mersha et al. (31) | Hypertension | lower quality |
| Li et al. (32) | Hypertension | Non-availability of data and non-response of the Corresponding author |
| Gemechu et al. (33) | Hypertension | lower quality |
| Rhee et al. (34) | Caffeine | smaller sample size |
| Greenwood et al. (35) | Caffeine | smaller sample size |
| Chen et al. (36) | Caffeine | smaller sample size |

| Rating | 16 | 15 | 14 | 13 | 12 | 11 | 10 | 9 | 8 | 7 | 6 | 5 | 4 | 3 | 2 | 1 | Items  Study |
| --- | --- | --- | --- | --- | --- | --- | --- | --- | --- | --- | --- | --- | --- | --- | --- | --- | --- |
| Critically low | Y | Y | Y | N | N | Y | N | Y | Y | N | Y | Y | Y | Y | Y | Y | Zhang Y |
| Critically low | N | PY | Y | PY | PY | Y | N | Y | PY | N | Y | Y | PY | Y | N | Y | Rahman M |
| Critically low | N | N | PY | N | N | Y | N | Y | PY | N | Y | Y | Y | Y | Y | Y | Pereira, P |
| Critically low | N | PY | PY | N | N | Y | N | Y | PY | N | Y | Y | Y | Y | Y | Y | Pereira, Ppd |
| Critically low | Y | Y | Y | N | PY | Y | N | Y | Y | N | Y | Y | PY | Y | N | Y | Liu, L |
| Critically low | Y | PY | PY | N | PY | Y | N | PY | PY | N | Y | Y | Y | Y | N | Y | Ghimire, U |
| Critically low | Y | PY | PY | PY | Y | Y | N | PY | PY | N | Y | Y | PY | Y | N | Y | Getaneh, T |
| Critically low | Y | PY | N | PY | PY | Y | N | Y | PY | N | N | N | PY | Y | N | Y | Engdaw, J |
| Critically low | Y | PY | PY | N | PY | Y | N | Y | Y | N | Y | Y | Y | Y | Y | Y | Dos Santos, J. F |
| Critically low | N | N | PY | Y | PY | Y | N | Y | PY | N | Y | Y | Y | Y | Y | Y | Cai, C |
| Critically low | PY | N | PY | N | N | Y | N | N | PY | N | Y | Y | PY | Y | N | Y | Hou J |
| Critically low | Y | PY | PY | PY | Y | Y | N | Y | PY | N | Y | Y | Y | Y | N | Y | Messerlian C |
| Critically low | PY | PY | Y | PY | Y | Y | N | PY | Y | N | Y | Y | Y | Y | N | Y | Jin F |

**Table 3. Assessing the Methodological Quality of Systematic Reviews using AMSTAR2**

Y: Yes; PY: Partial yes; N: No

1. Did the research questions and inclusion criteria for the review include the components of PICO? 2. Did the report of the review contain an explicit statement that the review methods were established prior to the conduct of the review and did the report justify any significant deviations from the protocol 3. Did the review authors explain their selection of the study designs for inclusion in the review? 4. Did the review authors use a comprehensive literature search strategy? 5. Did the review authors perform study selection in duplicate? 6. Did the review authors perform data extraction in duplicate? 7. Did the review authors provide a list of excluded studies and justify the exclusions? 8. Did the review authors describe the included studies in adequate detail? 9. Did the review authors use a satisfactory technique for assessing the risk of bias (RoB) in individual studies that were included in the review? 10. Did the review authors report on the sources of funding for the studies included in the review? 11. If meta-analysis was performed did the review authors use appropriate methods for statistical combination of results? 12. If meta-analysis was performed, did the review authors assess the potential impact of RoB in individual studies on the results of the meta-analysis or other evidence synthesis? 13. Did the review authors account for RoB in individual studies when interpreting/ discussing the results of the review? 14. Did the review authors provide a satisfactory explanation for, and discussion of, any heterogeneity observed in the results of the review? 15. If they performed quantitative synthesis did the review authors carry out an adequate investigation of publication bias (small study bias) and discuss its likely impact on the results of the review? 16. Did the review authors report any potential sources of conflict of interest, including any funding they received for conducting the review?

**Reference list of excluded systematic reviews**

1. McDonald SD, Han Z, Mulla S, Beyene J. Overweight and obesity in mothers and risk of preterm birth and low birth weight infants: Systematic review and meta-analyses. BMJ (Online). 2010;341(7765):187.

2. Rahman MM, Abe SK, Kanda M, Narita S, Rahman MS, Bilano V, et al. Maternal body mass index and risk of birth and maternal health outcomes in low- and middle-income countries: A systematic review and meta-analysis. Obesity Reviews. 2015;16(9):758-70.

3. Han Z, Mulla S, Beyene J, Liao G, McDonald SD. Maternal underweight and the risk of preterm birth and low birth weight: a systematic review and meta-analyses. International journal of epidemiology. 2011;40(1):65-101.

4. Liu Y, Wang Y, Wu Y, Chen X, Bai J. Effectiveness of the CenteringPregnancy program on maternal and birth outcomes: A systematic review and meta-analysis. International Journal of Nursing Studies. 2021;120.

5. Zheng BK, Li N. Methodological concerns about a systematic review and meta-analysis of maternal active smoking during pregnancy and low birth weight. Nicotine and Tobacco Research. 2018;21(6):850.

6. Palmer KT, Bonzini M, Harris EC, Linaker C, Bonde JP. Work activities and risk of prematurity, low birth weight and pre-eclampsia: an updated review with meta-analysis. Occupational and environmental medicine. 2013;70(4):213-22.

7. Casas M, Cordier S, Martínez D, Barros H, Bonde JP, Burdorf A, et al. Maternal occupation during pregnancy, birth weight, and length of gestation: Combined analysis of 13 European birth cohorts. Scandinavian Journal of Work, Environment and Health. 2015;41(4):384-96.

8. Cai C, Vandermeer B, Khurana R, Nerenberg K, Featherstone R, Sebastianski M, et al. The impact of occupational shift work and working hours during pregnancy on health outcomes: a systematic review and meta-analysis. American journal of obstetrics and gynecology. 2019;221(6):563-76.

9. Jarde A, Morais M, Kingston D, Giallo R, MacQueen GM, Giglia L, et al. Neonatal outcomes in women with untreated antenatal depression compared with women without depression: A systematic review and meta-analysis. JAMA psychiatry. 2016;73(8):826-37.

10. Huang H, Coleman S, Bridge JA, Yonkers K, Katon W. A meta-analysis of the relationship between antidepressant use in pregnancy and the risk of preterm birth and low birth weight. General Hospital Psychiatry. 2014;36(1):13-8.

11. Grote NK, Bridge JA, Gavin AR, Melville JL, Iyengar S, Katon WJ. A meta-analysis of depression during pregnancy and the risk of preterm birth, low birth weight, and intrauterine growth restriction. Archives of General Psychiatry. 2010;67(10):1012-24.

12. Grigoriadis S, Graves L, Peer M, Mamisashvili L, Tomlinson G, Vigod SN, et al. Maternal anxiety during pregnancy and the association with adverse perinatal outcomes: Systematic review and meta-analysis. Journal of Clinical Psychiatry. 2018;79(5).

13. Fekadu Dadi A, Miller ER, Mwanri L. Antenatal depression and its association with adverse birth outcomes in low and middle-income countries: A systematic review and meta-analysis. PloS one. 2020;15(1):e0227323.

14. Ding XX, Wu YL, Xu SJ, Zhu RP, Jia XM, Zhang SF, et al. Maternal anxiety during pregnancy and adverse birth outcomes: a systematic review and meta-analysis of prospective cohort studies. Journal of affective disorders. 2014;159:103-10.

15. Dadi AF, Miller ER, Mwanri L. Antenatal depression and its association with adverse birth outcomes in low and middleincome countries: A systematic review and meta-analysis. PloS one. 2020;15(1).

16. Liu Y, Zhuo L, Zhu B, He MY, Xu Y, Wang TT, et al. [Association between depression during pregnancy and low birth weight in neonates: a Meta analysis]. Zhongguo dang dai er ke za zhi = Chinese journal of contemporary pediatrics. 2017;19(9):994-8.

17. Patra J, Bakker R, Irving H, Jaddoe VW, Malini S, Rehm J. Dose-response relationship between alcohol consumption before and during pregnancy and the risks of low birthweight, preterm birth and small for gestational age (SGA)-a systematic review and meta-analyses. BJOG : an international journal of obstetrics and gynaecology. 2011;118(12):1411-21.

18. Haider BA, Olofin I, Wang M, Spiegelman D, Ezzati M, Fawzi WW. Anaemia, prenatal iron use, and risk of adverse pregnancy outcomes: systematic review and meta-analysis. BMJ (Clinical research ed). 2013;346:f3443.

19. Figueiredo ACMG, Gomes-Filho IS, Silva RB, Pereira PPS, Da Mata FAF, Lyrio AO, et al. Maternal anemia and low birth weight: A systematic review and meta-analysis. Nutrients. 2018;10(5).

20. Vergnes JN, Sixou M. Preterm low birth weight and maternal periodontal status: a meta-analysis. American journal of obstetrics and gynecology. 2007;196(2):135.e1-7.

21. Konopka T, Paradowska-Stolarz A. Periodontitis and risk of preterm birth and low birthweight - a meta-analysis. Ginekologia polska. 2012;83(6):446-53.

22. Khader YS, Ta'ani Q. Periodontal diseases and the risk of preterm birth and low birth weight: a meta-analysis. Journal of periodontology. 2005;76(2):161-5.

23. Corbella S, Taschieri S, Francetti L, De Siena F, Del Fabbro M. Periodontal disease as a risk factor for adverse pregnancy outcomes: a systematic review and meta-analysis of case-control studies. Odontology. 2012;100(2):232-40.

24. Corbella S, Taschieri S, Del Fabbro M, Francetti L, Weinstein R, Ferrazzi E. Adverse pregnancy outcomes and periodontitis: A systematic review and meta-analysis exploring potential association. Quintessence international (Berlin, Germany : 1985). 2016;47(3):193-204.

25. Porto ECL, Gomes Filho IS, Batista JET, Lyrio AO, Souza ES, Figueiredo A, et al. [Maternal periodontitis and low birth weight: systematic review and meta-analysis]. Ciencia & saude coletiva. 2021;26(suppl 3):5383-92.

26. Polyzos NP, Polyzos IP, Mauri D, Tzioras S, Tsappi M, Cortinovis I, et al. Effect of periodontal disease treatment during pregnancy on preterm birth incidence: a metaanalysis of randomized trials. American journal of obstetrics and gynecology. 2009;200(3):225-32.

27. Derakhshan A, Peeters RP, Taylor PN, Bliddal S, Carty DM, Meems M, et al. Association of maternal thyroid function with birthweight: a systematic review and individual-participant data meta-analysis. The lancet Diabetes & endocrinology. 2020;8(6):501-10.

28. Lutiger B, Graham K, Einarson TR, Koren G. Relationship between gestational cocaine use and pregnancy outcome: a meta-analysis. Teratology. 1991;44(4):405-14.

29. Hulse GK, English DR, Milne E, Holman CD, Bower CI. Maternal cocaine use and low birth weight newborns: a meta-analysis. Addiction (Abingdon, England). 1997;92(11):1561-70.

30. Addis A, Moretti ME, Ahmed Syed F, Einarson TR, Koren G. Fetal effects of cocaine: an updated meta-analysis. Reproductive toxicology (Elmsford, NY). 2001;15(4):341-69.

31. Mersha AG, Abegaz TM, Seid MA. Maternal and perinatal outcomes of hypertensive disorders of pregnancy in Ethiopia: systematic review and meta-analysis. BMC pregnancy and childbirth. 2019;19(1).

32. Li F, Wang T, Chen L, Zhang S, Chen L, Qin J. Adverse pregnancy outcomes among mothers with hypertensive disorders in pregnancy: A meta-analysis of cohort studies. Pregnancy hypertension. 2021;24:107-17.

33. Gemechu KS, Assefa N, Mengistie B. Prevalence of hypertensive disorders of pregnancy and pregnancy outcomes in Sub-Saharan Africa: A systematic review and meta-analysis. Womens Health. 2020;16.

34. Rhee J, Kim R, Kim Y, Tam M, Lai Y, Keum N, et al. Maternal Caffeine Consumption during Pregnancy and Risk of Low Birth Weight: A Dose-Response Meta-Analysis of Observational Studies. PloS one. 2015;10(7):e0132334.

35. Greenwood DC, Thatcher NJ, Ye J, Garrard L, Keogh G, King LG, et al. Caffeine intake during pregnancy and adverse birth outcomes: a systematic review and dose-response meta-analysis. European journal of epidemiology. 2014;29(10):725-34.

36. Chen LW, Wu Y, Neelakantan N, Chong MF, Pan A, van Dam RM. Maternal caffeine intake during pregnancy is associated with risk of low birth weight: a systematic review and dose-response meta-analysis. BMC medicine. 2014;12:174.
